# Use of the Pelvic C-Clamp to Mitigate Acute Respiratory Distress Syndrome in a Patient with an Unstable Sacral Fracture

**DOI:** 10.1155/2018/6412760

**Published:** 2018-02-18

**Authors:** Michael J. DeRogatis, Paul S. Issack

**Affiliations:** Department of Orthopaedic Surgery, New York-Presbyterian Hospital, New York, NY, USA

## Abstract

**Case:**

We present the case of a 21-year-old man who fell from a roof, sustaining a displaced sacral fracture with pelvic instability. He developed acute respiratory distress syndrome (ARDS) within 24 hours of injury. Placement of the pelvic C-clamp resulted in rapid resolution of pulmonary dysfunction, allowing for definitive internal fixation.

**Conclusion:**

The C-clamp is most commonly used to control hemorrhage in unstable posterior pelvic ring injuries. Our case demonstrates a rare use of the C-clamp to stabilize the posterior pelvis in a patient with an unstable sacral fracture and ARDS, to rapidly improve pulmonary function prior to definitive surgery.

## 1. Introduction

The pelvic C-clamp was introduced as a stabilization device to reduce and compress the posterior pelvis in patients with exsanguinating hemorrhage [1]. It has applications in resuscitating patients with unstable pelvic fractures with sacroiliac dissociation or vertical sacral fractures, where anterior external fixation may not adequately stabilize the posterior pelvis [2–5]. Stabilizing a vertical sacral fracture with pelvic instability can be difficult with pelvic binding/sheeting because of persistent motion at the fracture surfaces which can theoretically generate fat emboli and ARDS. We describe a case of a patient with an unstable sacral fracture who developed ARDS within 24 hours despite circumferential sheeting. Placement of a C-clamp resulted in a rapid restoration of pulmonary function, allowing for definitive open reduction and internal fixation (ORIF) of the sacrum. The patient was informed that data concerning the case would be submitted for publication, and the patient agreed.

## 2. Case Report

A 21-year-old previously healthy man was performing construction work when he slipped and fell from a 30-foot roof. He sustained a closed left-sided sacral fracture as well as right superior and inferior pubic ramus fractures. The patient initially had a pulse of 135 bpm, blood pressure of 104/63 mmHg, and hemoglobin of 11.7 g/dl. He received normal saline and 2 units of packed red cells. His pulse decreased to 104 bpm and blood pressure increased to 121/60 mmHg. Chest radiographs and FAST (focused assessment with sonography for trauma) examination were normal. At 12 hours, he remained hemodynamically stable (pulse 90 bpm, blood pressure 130/88 mmHg, and hemoglobin 10.1 g/dl).

**Figure 1:**
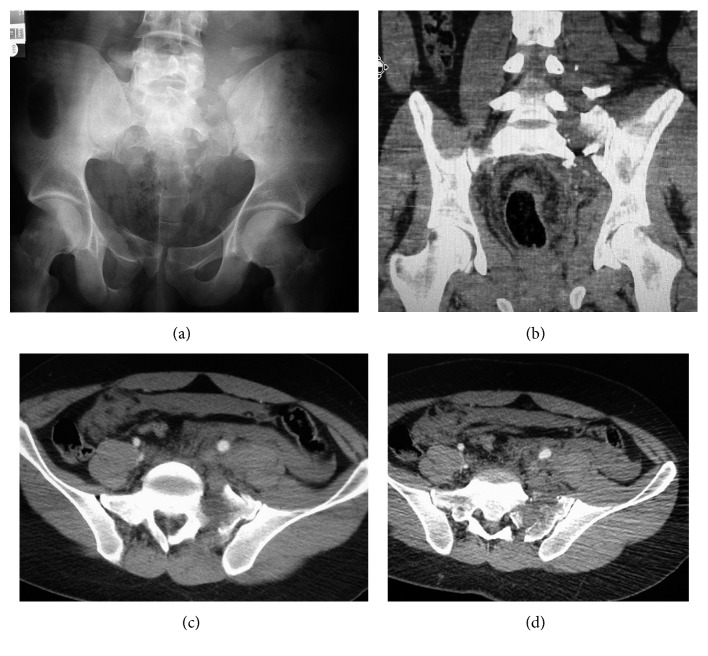
(a) Anteroposterior (AP) radiograph and (b) coronal CT cut demonstrating a comminuted Denis zone 2 sacral fracture with involvement of the sacral ala and fracture of the L4 and L5 transverse processes. (c, d) Axial CT cuts demonstrating the severe comminution involving the left sacral neuroforaminae.

On physical examination, the patient had severe pain in his left buttock. He was alert and oriented and had no other injuries. He had a left-sided foot drop. He had otherwise normal motor function including normal perianal sensation and rectal tone. AP pelvis radiographs and coronal and axial computed tomography (CT) cuts demonstrated left superior and inferior ramus fractures with a comminuted Denis zone 2 sacral fracture. There were fractures also laterally at the sacral alae (Figures 1(a)–1(d)). There were fractures of the left L4 and L5 transverse processes indicative of vertical instability, making this a type C pelvic ring injury. The patient was started on enoxaparin and sequential compression boots for deep venous thrombosis within 12 hours of admission. His fluid requirement was 1900 ml, 1560 ml, and 1090 ml of normal saline over the first 12, 24, and 48 hours, respectively.

While awaiting surgical clearance for fixation of the sacrum, the patient's pelvis was stabilized with circumferential sheeting which had been placed when the patient initially arrived. The patient was gently repositioned every 4 hours alternately placing a pillow under either the left or the right lumbar spine and buttock to diminish the risk of decubitus ulcer formation. Within 24 hours of admission, the patient demonstrated clinical and radiographic evidence of ARDS and required intubation and positive pressure ventilation. Bilateral infiltrates were noted on chest radiographs and chest CT. ORIF was delayed until the patient's pulmonary function resolved. However, 72 hours after admission, the patient still required significant positive pressure ventilation. One possible etiology of the pulmonary failure was thought to be multiple fat emboli released by motion at the sacral fracture site. Therefore, the decision was made to provide more rigid provisional skeletal stabilization using the pelvic C-clamp to, theoretically, reduce the fat embolic load to the lungs.

**Figure 2:**
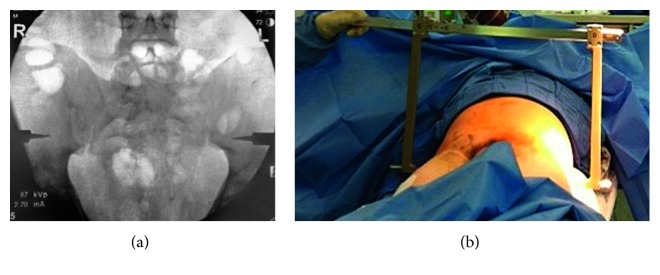
(a) AP fluoroscopic image after sacral reduction and provisional fixation with the spikes of the pelvic C-clamp embedded in the lateral ilium. (b) Clinical photograph of this patient with the pelvic C-clamp in place.

The patient was taken to the operating room intubated and under ventilator support. The patient was positioned supine on a radiolucent flat table. The pelvic sheet was removed. Posterior pelvic skin was intact with no evidence of ecchymosis or degloving injury. After prepping and draping, a starting point was identified by fluoroscopically locating the intersection of a line along the long axis of the femoral shaft and perpendicular to this line at the level of the anterior superior iliac spine. The side struts of the pelvic C-clamp (Synthes, PA) with attached spikes were passed through stab incision until the tips of the spikes contacted the lateral cortex of the flat portion of the ilium directly opposite the S1 body. Spike tips were hammered into the iliac cortex. The struts were manually compressed, and the outer screws were tightened to secure the frame. Screw tightening is performed under fluoroscopic guidance to avoid over compression of the zone 2 sacral fracture (Figures 2(a) and 2(b)).

**Figure 3:**
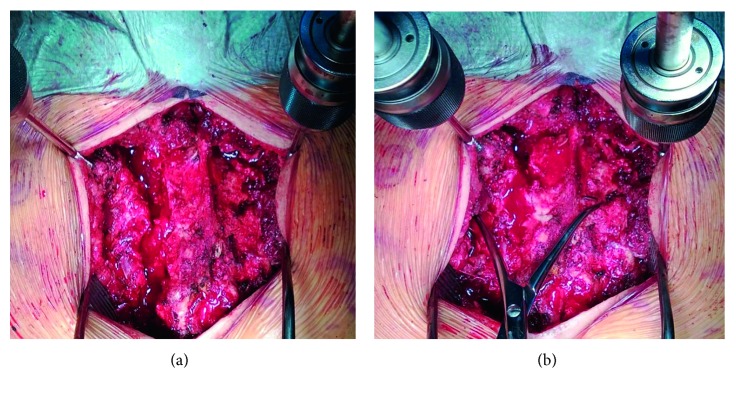
Intraoperative clinical photographs during definitive sacral ORIF. (a) Vertical zone 2 sacral fracture visible after irrigating and debriding the fracture site. Schantz pins attached to T-handle chucks placed in both posterior superior iliac spines are used as reduction tools. (b) Reduction of the sacral fracture maintained with a pelvic reduction forceps.

**Figure 4:**
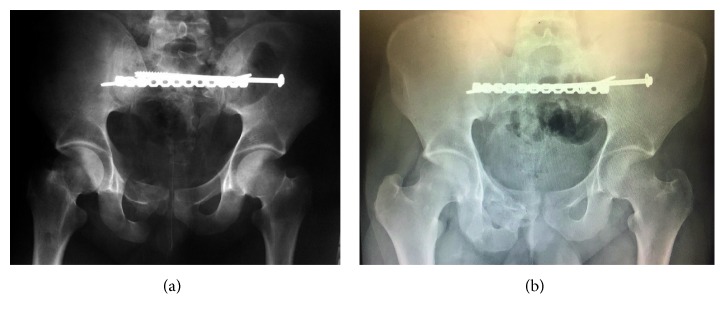
One-month (a) and one-year (b) postoperative AP radiographs.

Within 24 hours of C-clamp application, the patient's pulmonary function improved. The fraction of inspired oxygen and positive end-expiratory pressure decreased from 100% to 50% and 8 cm H_2_O to 5 cm H_2_O, respectively. The patient was kept intubated. Forty-eight hours after C-clamp application, the patient was returned to the operating room for definitive ORIF. The C-clamp was removed, and the patient was placed prone on a radiolucent flat table. Prepping and draping were performed, and the patient received intravenous antibiotics prior to skin incision. A midline skin incision was performed, with dissection carried out to the sacroiliac joints. The sacral fracture was debrided of interposing hematoma and periosteum. Reduction was performed using a pelvic reduction forceps placed on the spinous process and lateral ilium, and Schanz pins placed in the posterior superior iliac spines (Figures 3(a) and 3(b)). Under fluoroscopic imaging, a sacroiliac screw was placed in the left sacrum, S1 body and extending into the right sacrum. Care was taken not to over compress the fracture site. A reconstruction plate was added posteriorly as a tension band plate (Figure 4(a)).

The patient was extubated within 12 hours after surgery. He was maintained on 20 lbs foot flat weight bearing for 3 months. At one-year postop, the patient ambulates without assistive devices (Figure 4(b)). He has no pain and does not use an ankle foot orthosis, although he continues to lack active ankle dorsiflexion.

## 3. Discussion

In this case, we used the C-clamp to help manage ARDS secondary to an unstable sacral fracture. Continued motion at the fracture surfaces of a vertical sacral fracture can theoretically result in the generation of fat emboli, which may contribute to the development of ARDS [6, 7]. Therefore, obtaining rigid temporary skeletal stabilization of the sacral fracture with a C-clamp may reduce the production of fat emboli and progression of ARDS. It is important to note that severe trauma and pulmonary contusion can cause ARDS without the development of fat embolism syndrome, and an unstable pelvis may not be necessary for the pathogenesis. It is possible that this patient suffered from pulmonary contusion at the time of his fall, and the infiltrates seen on chest CT may have been present at the time of initial presentation.

The device is mainly used to control hemorrhage in rotationally and vertically unstable pelvic fractures with disruption of the posterior ring (Tiles B and C) [1–5]. It has also been used as to reduce sacral fractures and sacroiliac fracture dislocations [8–10]. Ertel and colleagues reported on 20 patients with unstable pelvic fractures (Tile B or C) in hemorrhagic shock [2]. All patients underwent immediate stabilization with a C-clamp. Mortality rate was 25%; four patients died from exsanguination within 9 hours [2]. Sadri and colleagues reviewed 14 patients with unstable pelvic ring fractures (Tile B or C) and hemorrhagic shock treated with immediate C-clamp stabilization. Thirty-six percent of these patients required subsequent arterial embolization to control bleeding. Mortality rate was 14% [5].

Could C-clamp fixation have been bypassed for definitive ORIF in our patient? In a patient with pulmonary injury, damage control principles aim to minimize risk of a “second hit,” such as early total care surgery, that can worsen systemic inflammation and pulmonary function [11–13]. Vallier and colleagues noted that, in 124 patients with severe pulmonary injury and pelvic or acetabular fractures, either early or delayed open reduction and internal fixation resulted in increased pulmonary complications (32% versus 10%, *p* < 0.0001) and ARDS (20% versus 3.3%, *p* < 0.0001), compared with patients with minimal chest injury [6]. Engstrom and colleagues retrospectively reviewed the incidence of pulmonary failure in patients with pelvic fractures admitted to the intensive care unit. They found that lung injury was associated with the development of respiratory failure during or after surgical stabilization of the pelvis. The authors suggest that the definitive surgical procedure may act as a “second hit” and deteriorate lung function [14]. The C-clamp, analogous to an external fixator, can provide rapid skeletal stabilization and provide damage control, mitigating the risk of the “second hit”. Furthermore, inappropriate mechanical ventilation during surgery on the sacrum as well as pulmonary emboli resulting from the necessary manipulation that occurs when debriding and reducing the fracture surfaces may increase postoperative respiratory complications [15]. We used the C-clamp to provisionally stabilize the sacrum and posterior pelvis in the hope that lung function would improve, thus making definitive surgery safer from a pulmonary standpoint.

Application of the pelvic C-clamp should be performed by experienced trauma surgeons [16, 17]. Complications with C-clamp placement include superior gluteal artery and sciatic nerve injury if the spikes are inserted too distally [16, 18, 19]. Anterior placement can cause intrapelvic organ injury [18, 19]. External rotation of the hemipelvis should be manually corrected before spike placement; otherwise, the risk of spike malpositioning and hemipelvic dislocation increases [20].

It is possible that the normal inflammatory course occurred, and the patient began to recover pulmonary function 72 hours after developing ARDS. However, the normal course of ARDS can take several days to weeks [7, 14]. Furthermore, the patient demonstrated dramatic improvement in pulmonary function within 24 hours after C-clamp placement. This rapid improvement of pulmonary function suggests that the C-clamp contributed at least in part to this recovery.
